# Moxibustion and Acupuncture Ameliorate Crohn's Disease by Regulating the Balance between Th17 and Treg Cells in the Intestinal Mucosa

**DOI:** 10.1155/2015/938054

**Published:** 2015-08-04

**Authors:** Chen Zhao, Chunhui Bao, Jing Li, Yifang Zhu, Siyao Wang, Ling Yang, Yin Shi, Huirong Liu, Chuanzi Dou, Guanghong Ding, Xiaomei Wang, Huangan Wu

**Affiliations:** ^1^Key Laboratory of Acupuncture-Moxibustion and Immunological Effects, Shanghai University of Traditional Chinese Medicine, Shanghai 200030, China; ^2^Department of Acupuncture-Moxibustion, Yueyang Hospital of Integrated Traditional Chinese and Western Medicine, Shanghai University of Traditional Chinese Medicine, Shanghai 200437, China; ^3^Department of Acupuncture-Moxibustion, Shengzhou Hospital of Traditional Chinese Medicine, Zhejiang 312400, China; ^4^Department of Mechanics and Engineering Science, Fudan University, Shanghai 200433, China; ^5^Shanghai Research Center of Acupuncture and Meridian, Shanghai 201203, China

## Abstract

Previous studies have demonstrated that acupuncture is beneficial to patients with Crohn's disease (CD), but the mechanism underlying its therapeutic effects remains unclear. To identify the mechanism by which acupuncture treats CD, the balance between Th17 and Treg cells was assessed in CD patients. In this study, Ninety-two CD patients were randomly and equally assigned to a treatment group that were treated with herb-partitioned moxibustion and acupuncture or a control group with wheat bran-partitioned moxibustion and superficial acupuncture. The effect of these treatments on Th17 and Treg cells and their related molecular markers in the intestinal mucosa were detected before (week 0) and after (week 12) treatment. The results suggested that the ratio of Th17 and Treg cells was significantly decreased after treatment and that the levels of IL-17 and ROR*γ*t in the intestinal mucosa were obviously reduced, while the expression of FOXP3 was increased after treatment in both groups. In the treatment group, the expression of these molecules was more markedly regulated than the control group. In conclusion, moxibustion and acupuncture have been shown to regulate the ratio of Th17 and Treg cells in the intestinal mucosa of CD patients and restore the balance between these immune cell subsets.

## 1. Introduction

Crohn's disease (CD) is a nonspecific granulomatous inflammatory bowel disease (IBD) with an unknown etiology. The main clinical manifestations are recurrent episodes of abdominal pain, diarrhea, and weight loss and are often complicated with abdominal mass, anal fistula, and intestinal obstruction. The disease is characterized with a long duration and frequent recurrence and cure difficulty. With the disease incidence increasing over years, it has become one of the most common diseases in the intestinal tract [1]. Young adults are the main implicated populations and severely suffer in terms of their health condition and quality of life [2, 3]. Current treatments in modern medicine include aminosalicylates, corticosteroids, and immunomodulators that are effective in the management of the acute symptoms of this disease; however, the side effects accompanied with long periods of usage have limited their long-term use. The biological agent of anti-TNF-*α* offers an alternative treatment option, but its high price renders it unavailable to many patients. Therefore, new forms of treatment are urgently needed in clinical practice.

Acupuncture and moxibustion are an important component of Traditional Chinese Medicine (TCM) with a history of over 4,000 years and have gradually been accepted by many countries worldwide. They are widely used in the clinical treatment of various diseases, especially gastrointestinal diseases, such as Crohn's disease [4, 5], ulcerative colitis [6, 7], irritable bowel syndrome [8–10], and functional dyspepsia [11, 12]. Our clinical study has previously demonstrated that moxibustion combined with acupuncture is not only effective in treating CD with an improvement of patients' CDAI scores and their quality of life, and it also reduces CRP levels and increases hemoglobin levels in patients [4]. However, the biological mechanisms underlying the treatment of CD by moxibustion and acupuncture have not been fully elucidated.

Th17 and Treg cells are two recently discovered subsets of T lymphocytes. Research has shown that an imbalance between Th17 and Treg cells is involved in the development of CD [13, 14]. Th17 cells play important roles in mediating inflammatory responses. Treg cells inhibit the proliferation of effector T cells and their reaction to autoantigens, thus controlling the strength of immune responses elicited by effector T cells and limiting the severity of associated tissue damage [15, 16]. Th17 and Treg cells are closely related during differentiation because they share the same precursor population. A balanced production of both cell types is critical to the maintenance of intestinal homeostasis. The purpose of the present study was to determine the anti-inflammatory mechanism of moxibustion and acupuncture in the treatment of CD by assessing the effects of moxibustion and acupuncture on the ratio of Th17 and Treg cells and the expression of key related molecules (forkhead box P3 (FOXP3), retinoid-related orphan receptor gamma (ROR*γ*t), and interleukin-17 (IL-17)) in the intestinal mucosa of patients with CD.

## 2. Materials and Methods

### 2.1. Study Design

#### 2.1.1. Participants

From January 2010 to April 2013, patients with Crohn's disease (CD) were recruited for this study at the following hospitals: the outpatient clinic for inflammatory bowel disease of Shanghai Institute of Acupuncture and Meridian at Shanghai University of TCM, the Endoscopy Center of Zhongshan Hospital at Fudan University, the Shuguang Hospital at Shanghai University of TCM, and the Yueyang Hospital of Integrated Traditional Chinese and Western Medicine at Shanghai University of TCM. The diagnosis of CD was confirmed in all patients by imaging, endoscopic, and histopathological examinations [4].

Inclusion criteria were as follows: patients with mild to moderate CD (CDAI between 151 and 350), without taking any medication or taking only salicylates and/or prednisone (dose ≤ 15 mg treatment lasting at least one month) and without the use of immunosuppressants or anti-TNF-*α* biological agents within the past 3 months. Exclusion criteria were as follows: Women who were pregnant or breast-feeding; patients with severe diseases in the heart, brain, liver, kidney, or hematopoietic system; patients with any mental illness.

#### 2.1.2. Interventions

Ninety-two patients with active CD were randomly assigned at a 1 : 1 ratio to a treatment group and a control group. Patients in the treatment group were treated with herb-partitioned moxibustion combined with acupuncture: moxibustion was performed on the Tianshu (ST25, bilateral), Qihai (CV6), and Zhongwan (CV12) acupoints; and acupuncture was performed at the Zusanli (ST36), Shangjuxu (ST37), Sanyinjiao (SP6), Taixi (KI3), Gongsun (SP4), and Taichong (LR3) acupoints. For herb-partitioned moxibustion, the herbal cake contained* Coptis chinensis*, Radix* Aconiti Lateralis*,* Cortex Cinnamomi*, Radix Aucklandiae,* Flos carthami*,* Salvia miltiorrhiza*, and* Angelica sinensis* as the main ingredients. All these herbs were ground into a fine powder, mixed with maltose and warm water to make into a thick paste, and pressed with a mold into herbal cakes with a diameter of 28 mm and thickness of 5 mm. Pure refined moxa sticks that were 16 mm tall with a diameter of 17 mm (“Hanyi,” Nanyang, China) were placed on top of the herbal cakes for moxibustion. In each session, two moxa sticks were used per acupoint. Disposable sterile stainless steel needles (0.30 mm in diameter, 40 mm or 25 mm long, “Hwato,” Suzhou, China) were used for acupuncture. After local routine disinfection, the needles were directly inserted up to 20–30 mm into the skin to elicit a* de-qi* sensation. The needles were then left in place for 30 minutes. Moxibustion and acupuncture were simultaneously performed once every other day (three times a week) for a total of 36 sessions (12 weeks). Patients in the control group were treated with wheat-bran-partitioned moxibustion combined with superficial needle puncture at nonmeridian, nonacupoint sites. For moxibustion, wheat bran was used instead of herbal powder to make wheat bran cakes of the same size, but the acupoints and the moxibustion method used were the same as the treatment group. The same acupuncture needles were used for superficial needle puncture. Nonmeridian, nonacupoint zones located 1-2 cm away from the acupoints of the treatment group were selected for superficial needle puncture and the same number of sites was inserted with needles. After local routine disinfection, the needles were directly inserted only 1-2 mm into the skin, without eliciting a* de-qi* sensation. These needles were left in place for 30 min. Wheat-bran-partitioned moxibustion and superficial needle puncture were performed at the same time. Total numbers of sessions were the same as in the treatment group.

#### 2.1.3. Sample Collection

Ten patients were randomly selected from each group for the present study. At the time of recruitment (week 0) and at the end of treatment (week 12), these patients received colonoscopic examination and intestinal biopsies were taken from the ileocecal region. Four biopsies were taken each time and stored in formaldehyde or liquid nitrogen for future detection.

#### 2.1.4. Ethical Approval and Trial Registration

This clinical trial was approved by the Ethics Committee at the Yueyang Hospital of Integrated Traditional Chinese and Western Medicine at Shanghai University of TCM. All subjects signed the informed consent forms. This trial was registered at the following website: http://clinicaltrials.gov/ (NCT01697761).

### 2.2. Experimental Methods 

#### 2.2.1. HE Staining

The tissue biopsies were processed in an automated tissue processing machine and then embedded into paraffin. Then 4 *μ*m continuous sections were cut, deparaffinized by two changes of xylene, each lasting 20 min, and then rehydrated through 100%, 95%, 85%, and 75% ethanol, each round lasting 3 min. The sections were stained with hematoxylin for 1 min, differentiated in a 1% hydrochloric acid-ethanol solution (one part of hydrochloric acid with 100 parts of 70% ethanol), and then stained in a 0.5% aqueous solution of eosin for 5 min. The sections were examined and scored under a microscope.

#### 2.2.2. Immunofluorescence Staining

An immunofluorescence double labeling technique was used to determine the ratio of Th17 and Treg cells in the intestinal mucosa. Deparaffinized sections were washed three times in 0.01 M PBS (pH 7.2–7.6) for 5 min each and then heated to 92–98°C for antigen retrieval. Sections were blocked with 10% goat serum for 30 min and then incubated with rabbit anti-human IL-17 and rabbit anti-human FOXP3 (both at 1 : 100, Abcam, U.K.) in a humidified chamber overnight at 4°C. Sections were then incubated with diluted secondary antibodies and mounted with an antifluorescence quenching agent or glycerol: 0.01 M PBS (1 : 1). The sections were photographed under a fluorescent microscope (BX53 Olympus). IL-17 served as the marker for Th17 cells and was labeled with Cy3, and FOXP3 served as the marker for Treg cells and was labeled with FITC. Nuclei were stained by DAPI. Three microscopic fields were randomly chosen from each slide and analyzed with the Image Pro Plus (IPP) analysis system to obtain fluorescence intensity for the positive staining.

#### 2.2.3. Real-Time RT-PCR

Total RNA was extracted from tissues using Trizol (Invitrogen, U.S.) and the RNA content and quality were determined with Nanodrop. Then 4 mg of total RNA was reverse-transcribed into cDNA with a reverse transcription kit (Thermo, U.S.), and 100 ng cDNA was used as template for the real-time PCR reaction. Primers were added at 200 nM into the reactions, and SYBR Green was used for detection. Real-time PCR was run in an ABI7300 instrument (running ABI Prism 7300 SDS Software). The relative expression levels of IL-17, ROR*γ*t, and FOXP3 mRNAs are presented as 2^−ΔCt^ (ΔCt = Ct value of the target gene − Ct reference gene value). Primers are listed in Table 1.

#### 2.2.4. Immunohistochemical Staining

The Envision method was used for immunohistochemistry. Sections were deparaffinized, rehydrated, antigen-retrieved with sodium citrate buffer, and blocked with 20% normal goat serum in a humidified chamber at 37°C for 30 min and then incubated with primary antibodies (mouse anti FOXP3, 1 : 100, Abcam; rabbit anti-IL-17 and rabbit anti-ROR*γ*t, 1 : 100, Abcam) at 4°C overnight. Sections were then incubated with HRP-labeled secondary antibodies (rabbit anti-mouse IgG, HRP Conjugated for FOXP3, goat anti-rabbit IgG, HRP Conjugated for IL-17 and ROR*γ*t) at 37°C for 60 min, followed by DAB for color development. Sections were then counterstained with hematoxylin, differentiated in a 0.1% hydrochloric acid-ethanol solution, and mounted with a neutral resin. Three microscopic fields were randomly chosen from each slide and analyzed with the IPP image analysis system to obtain integrated optic density (IOD) for the positive staining.

### 2.3. Statistical Methods

Statistical analysis was performed using the SPSS13.0 software package. Quantitative data with normal distribution and homogeneous variances are represented as mean ± standard deviation ($\overset{-}{x}\pm s$). Comparisons within groups were conducted with paired *t*-tests, while comparisons between groups were conducted with two-sample *t*-tests. Quantitative data that were not normally distributed or showed heterogeneity of variance are represented as median (P25–P75). Comparisons within groups were conducted using paired-sample Wilcoxon signed rank tests, and comparisons between groups were conducted using nonparametric Mann-Whitney *U* tests. All tests were two sided with significance set to *α* = 0.05, *P* < 0.05 and was considered statistically significant.

## 3. Results

After the treatments, the CDAI scores of patients in the treatment group (*n* = 10) decreased by 111.15 ± 54.74, while those of patients in the control group (*n* = 10) only decreased by 40.51 ± 31.36. The values decreased between the two groups were significantly different (*P* = 0.002).

### 3.1. Pathological Changes in the Intestinal Mucosa

**Figure 1:**
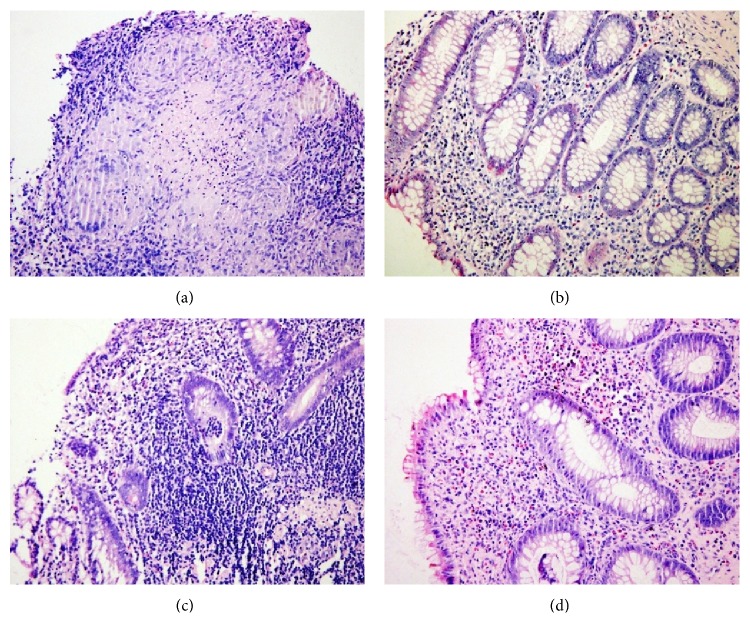
HE staining images of the intestinal mucosa from both groups of patients (a) before and (b) after treatment from patients in the treatment group and (c) before and (d) after treatment from patients in the control group (200x).

Before treatment, the intestinal biopsies from both groups showed pathological characteristics typical of Crohn's disease. A large number of giant, multinucleated cells were present in granulomas, the centers of which contained necrotic cells and infiltrated inflammatory cells. The mucosa epithelium was damaged or missing, and the lamina propria was infiltrated by many lymphocytes. Also visible were structures resembling lymphoid follicles. The intestinal glands were damaged and disorganized, containing necrotic epithelial cells, infiltrated inflammatory cells, and multinucleated giant cells. After the moxibustion and acupuncture treatment, the intestinal mucosal epithelium was intact, the intestinal glands were reorganized, and less inflammatory cells infiltrated. After control treatment, the mucosal epithelium also became intact, but inflammatory cell infiltration was still visible and the glands were less organized than the treatment group (Figure 1).

### 3.2. Th17 and Treg Cells in the Intestinal Mucosa

**Figure 2:**
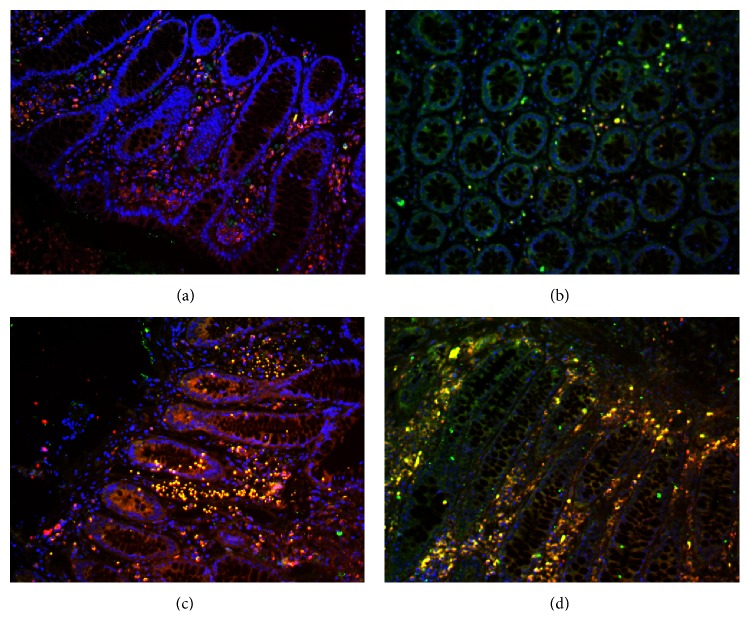
Th17 and Treg cells in the intestinal mucosa. Th17 (red) and Treg (green) cells were detected by immunofluorescence (a) before and (b) after treatment from patients in the treatment group and (c) before and (d) after treatment from patients in the control group (200x).

**Figure 3:**
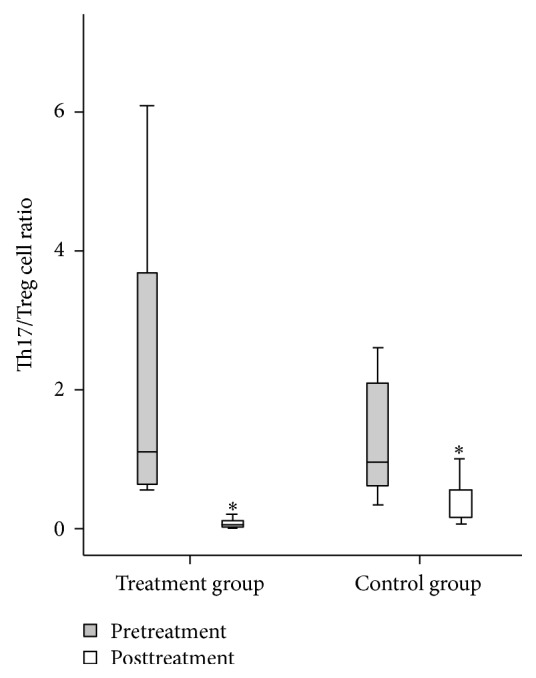
The ratio of Th17/Treg cells in the intestinal mucosa of both groups of patients before and after treatment. Comparison within groups, ^∗^ *P* < 0.05.

Before the treatments, a large number of IL-17 + T cells in the intestinal mucosa infiltrated in both groups, and the IL-17 + T cells were mainly located in the lamina propria. After treatment, there were significantly more FOXP3 + Treg cells in both cases but significantly fewer IL-17 + T cells. Treg cells were localized mainly in the cortex and lamina propria. In both groups of patients, the ratio of Th17/Treg was significantly lower after treatment (*P* < 0.01 and *P* < 0.05). Comparisons between the two groups either before or after treatment showed no significant difference in the Th17/Treg ratio (*P* > 0.05) (Figures 2-3).

### 3.3. IL-17 Protein and mRNA Expression in the Intestinal Mucosa

#### 3.3.1. IL-17 Protein Expression in the Intestinal Mucosa

**Figure 4:**
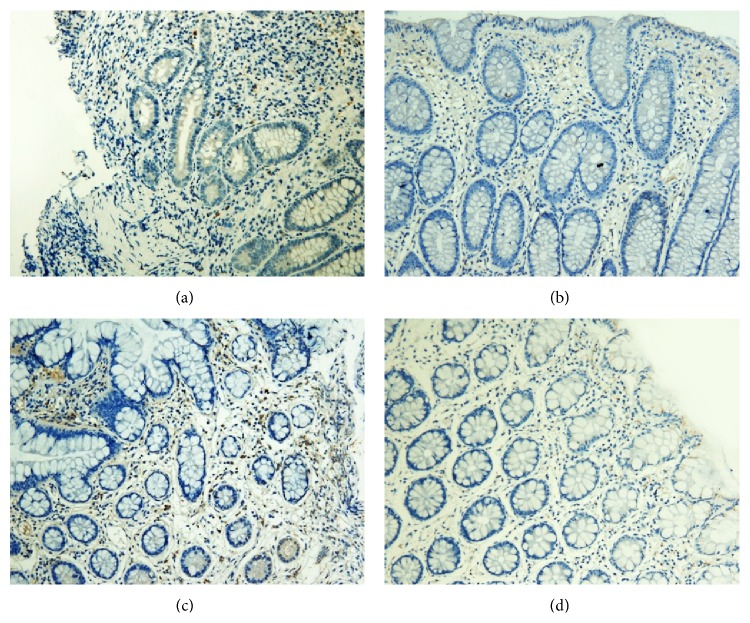
Expression of IL-17 in the intestinal mucosa (a) before and (b) after treatment from patients in the treatment group and (c) before and (d) after treatment from patients in the control group (200x).

**Figure 5:**
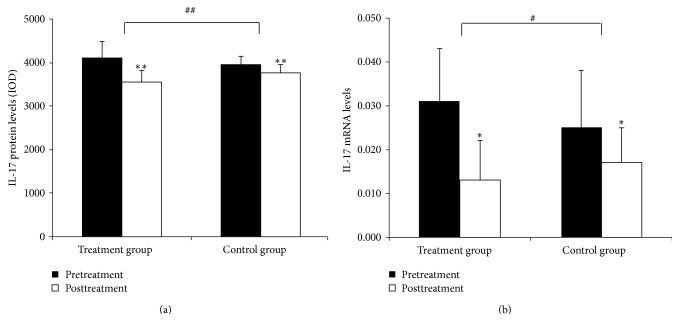
Quantitation of IL-17 mRNA and protein levels in the intestinal mucosa. (a) Levels of IL-17 protein in the intestinal mucosa. (b) Levels of IL-17 mRNA in the intestinal mucosa. Comparison within groups, ^∗^ *P* < 0.05, ^∗∗^ *P* < 0.01; comparison between groups, ^#^ *P* < 0.05, ^##^ *P* < 0.01.

IL-17 proteins were mainly found in the lamina propria. IL-17 levels were significantly lower after treatment in both groups (*P* < 0.01 for both). The decrease in IL-17 protein levels was significantly greater in patients in the treatment group (*P* < 0.01) (Figures 4–5(a)).

#### 3.3.2. IL-17 mRNA Expression in the Intestinal Mucosal

IL-17 mRNA levels were significantly lower after treatment in both groups (*P* < 0.05 for both). The decrease in IL-17 mRNA levels was significantly greater in patients in the treatment group (*P* < 0.01) (Figure 5(b)).

### 3.4. ROR*γ*t Protein and mRNA Expression in the Intestinal Mucosa

#### 3.4.1. ROR*γ*t Protein Expression in the Intestinal Mucosa

**Figure 6:**
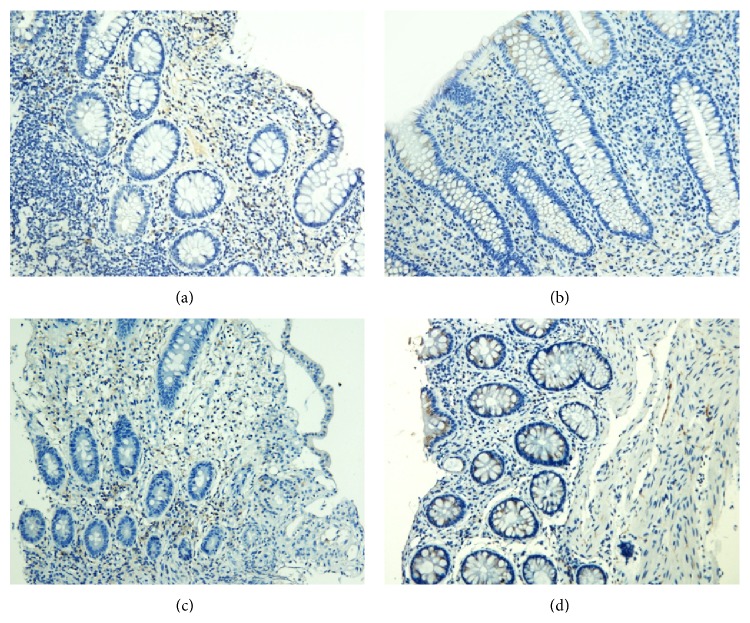
Expression of ROR*γ*t in the intestinal mucosa (a) before and (b) after treatment from patients in the treatment group and (c) before and (d) after treatment from patients in the control group (200x).

**Figure 7:**
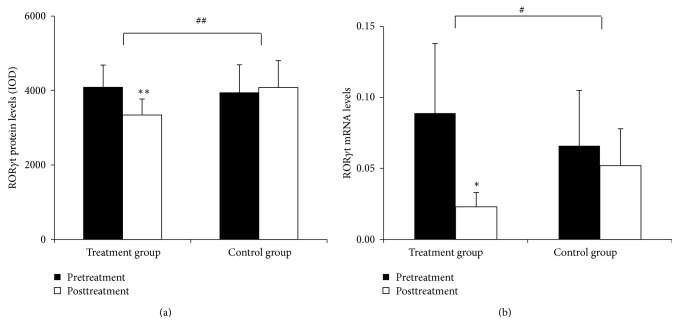
Quantitation of ROR*γ*t mRNA and protein levels in the intestinal mucosa. (a) Levels of ROR*γ*t protein in the intestinal mucosa. (b) Levels of ROR*γ*t mRNA in the intestinal mucosa. Comparison within groups, ^∗^ *P* < 0.05, ^∗∗^ *P* < 0.01; comparison between groups, ^#^ *P* < 0.05, ^##^ *P* < 0.01.

Positive staining for ROR*γ*t protein was mainly found in the lamina propria. ROR*γ*t protein levels were significantly lower in patients in the treatment group (*P* < 0.01), but these values were not significantly different in patients in the control group (*P* > 0.05). Comparisons between the two groups showed that the treatment group had a significantly greater response in reducing ROR*γ*t expression (*P* < 0.01) (Figures 6–7(a)).

#### 3.4.2. ROR*γ*t mRNA Expression in the Intestinal Mucosa

In the treatment group, ROR*γ*t mRNA levels were significantly decreased after the treatment (*P* < 0.05), but levels were only slightly lower in the control group (*P* > 0.05). Comparisons between the two groups showed that the therapy in the treatment group had a significantly more pronounced effect to downregulate ROR*γ*t mRNA expression (*P* < 0.01) (Figures 6–7(a)).

### 3.5. FOXP3 Protein and mRNA Expression in the Intestinal Mucosa

#### 3.5.1. FOXP3 Protein Expression in the Intestinal Mucosa

**Figure 8:**
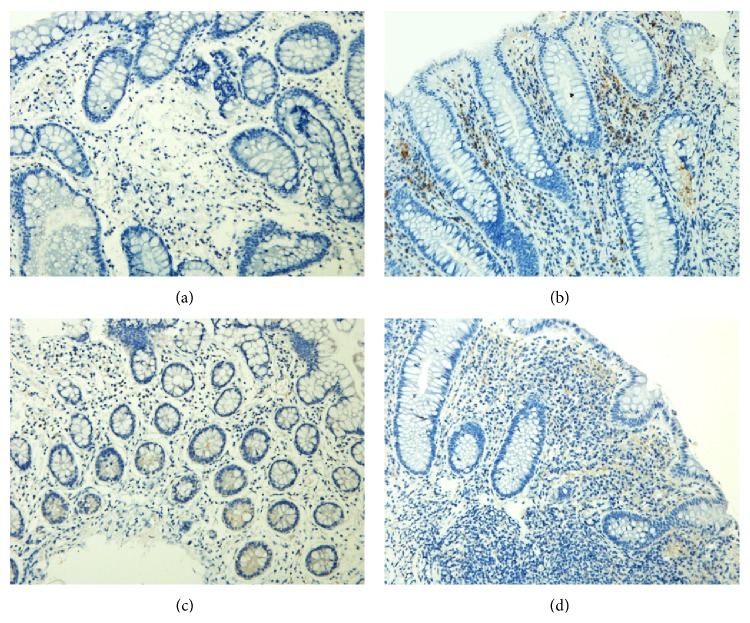
Expression of FOXP3 in the intestinal mucosa (a) before and (b) after treatment from patients in the treatment group and (c) before and (d) after treatment from patients in the control group (200x).

**Figure 9:**
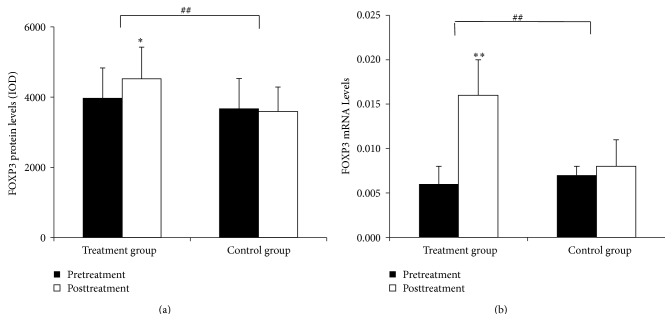
Quantitation of FOXP3 mRNA and protein levels in the intestinal mucosa. (a) Levels of FOXP3 protein in the intestinal mucosa. (b) Levels of FOXP3 mRNA in the intestinal mucosa. Comparison within groups, ^∗^ *P* < 0.05, ^∗∗^ *P* < 0.01; comparison between groups, ^#^ *P* < 0.05, ^##^ *P* < 0.01.

FOXP3 protein was mainly detected in the epithelium and lamina propria. In the treatment group, FOXP3 protein levels were significantly higher after the treatment (*P* < 0.05) but were not significantly different in the control group (*P* > 0.05). Comparisons between the two groups showed that the therapy in the treatment group has a significantly more pronounced increase in FOXP3 expression (*P* < 0.01) (Figures 8–9(a)).

#### 3.5.2. FOXP3 mRNA Expression in the Intestinal Mucosa

In the treatment group, FOXP3 mRNA levels were significantly higher after the treatment (*P* < 0.01), but the difference was not significant in the control group (*P* > 0.05). Comparisons between the two groups showed that the therapy in the treatment group had a significantly greater effect in upregulating FOXP3 mRNA expression (*P* < 0.01) (Figure 9(b)).

## 4. Discussion

It has demonstrated that moxibustion and acupuncture are effective and safe in treating CD, but their therapeutic mechanism has not been fully elucidated. The results of the present study showed that moxibustion and acupuncture inhibited the protein and mRNA expression of IL-17 and ROR*γ*t but induced the protein and mRNA expression of FOXP3 in the intestinal mucosa of CD patients. Moxibustion and acupuncture restored the balance between intestinal Th17 and Treg cells in CD patients, thus relieving intestinal inflammation in CD patients by regulating the differentiation of these two cell subsets.

The subjects of this study were from a previous randomized, controlled clinical trial [4]. Patients in the treatment group were treated with acupuncture combined with herb-partitioned moxibustion, and patients in the control group were treated with superficial needle puncture at nonmeridian, nonacupoint sites combined with wheat bran-partitioned moxibustion as the placebo treatment. The results of this trial showed that the moxibustion and acupuncture in the treatment group to be significantly more effective than the control group in relieving the symptoms and intestinal inflammation in CD patients. The moxibustion and acupuncture treatment significantly decreased the CDAI scores of patients and increased their IBDQ scores which is associated with quality of life. In addition, the moxibustion and acupuncture treatment significantly reduced C-reactive protein levels and increased hemoglobin levels of patients. Moxibustion and acupuncture in the treatment group had significantly more pronounced effects than the control group for all these measurements, suggesting that moxibustion and acupuncture have a significant therapeutic effect [4]. Ten patients from each group were randomly selected to undergo colonoscopy and intestinal biopsy, and results showed that the moxibustion and acupuncture treatment outperformed the control treatment in reducing inflammation in the intestinal mucosa. Therefore, it led us to further investigate the mechanism through which moxibustion and acupuncture modulate the inflammation in the intestinal mucosa.

An imbalance between Th17 and Treg cells constituted a key step in the disruption of intestinal homeostasis and is one of the major contributors to the development and progression of CD [17]. The main function of Th17 cells is to secrete IL-17 and other proinflammatory cytokines, which in turn induce the migration of neutrophils towards sites of infection to elicit an inflammatory response. IL-17A can exert a strong proinflammatory effect. It can also enhance cell permeability and promote the production of other proinflammatory cytokines and chemokines. Various types of cells, such as epithelial cells, endothelial cells, and fibroblasts all possess abundant IL-17 receptors (IL-17R) on the cell surface [18, 19]. Research has shown that Th17 cells are the major contributor to inflammation in CD. The intestinal mucosa of CD patients is infiltrated by a large number of Th17 cells. The number of Th17 cells in the intestinal mucosa and the level of IL-17, which is secreted by Th17 cells, in the intestinal mucosa and serum, were significantly higher in patients with CD than in healthy people. They were also higher in patients with acute disease than in patients whose disease is in remission [20, 21]. The level of IL-17A is positively correlated with the severity of CD, while IL-17A deficiency is associated with resistance to colitis development [22], suggesting that IL-17A promotes colitis. ROR*γ*t is an important transcription factor regulating Th17 cell differentiation. Th17 cells express inflammatory cytokines, including IL-17A, IL-17F, IL-21, and IL-23, which can initiate innate and adaptive immune responses [17].

Moreover, studies have suggested that a reduction in the number or function of Treg cells might be a major cause of the pathogenesis of Crohn's disease [23, 24]. Tregs can suppress intestinal mucosal inflammation induced by innate or acquired immunity. FOXP3 and IL-2 promote the differentiation of Treg cells. Reduced numbers of Treg cells and deficiency in their function can cause damage in the intestinal mucosa, resulting in CD [25]. Tregs suppress Th effector cell activity by secreting IL-10 and/or TGF-*β*1, and IL-10 inhibits the proliferation of Th cells and their production of inflammatory factors. A clinical study showed that the number of Tregs in the peripheral blood and intestinal mucosa was significantly lower in CD patients than in healthy subjects, but the function of Tregs was not altered [26]. FOXP3 is specifically expressed in Treg cells. Its expression was significantly lower in CD patients than in healthy subjects and also lower in patients with acute diseases than those in remission [27]. Although Treg cells can dampen intestinal mucosal inflammation effectively in patients with CD, in the presence of IL-6 and/or IL-23, Tregs can differentiate into Th17 cells, which then accelerate the inflammatory process [28]. It was shown that the ratios of Treg/Th17 cells in the peripheral blood and intestinal mucosa were significantly lower in CD patients [29]. As such, restoring the balance between these cells is essential for the treatment of intestinal inflammation in CD.

The present study showed that moxibustion and acupuncture can regulate and restore the balance between Th17 and Treg cells in intestinal mucosa of patients with CD. The moxibustion and acupuncture treatment reduced the number of Th17 cells and inhibited the expression of Th17-related molecules IL-17 and ROR*γ*t in the intestinal mucosa. It also increased the number of Treg cells and the expression of Treg-specific transcription factor FOXP3, thus restoring the ratio of the two cell types. The control treatment also reduced intestinal mucosal inflammation in CD patients to some extent, but it was not as effective as the moxibustion and acupuncture treatment. The regulatory effect of moxibustion and acupuncture on the balance between Th17 and Treg cells has been demonstrated in animal models of autoimmune encephalitis [30, 31]. Therefore, we speculated the following: (1) moxibustion and acupuncture directly regulate the differentiation of naive T cells in intestinal mucosa of patients with CD, which makes T cells differentiate towards the direction of Treg cells, resulting in an increase in the number of Treg cells, whereas the number of Th17 cells is correspondingly reduced; (2) moxibustion and acupuncture inhibit IL-17 and ROR*γ*t expression at the protein and mRNA levels and promote the expression of FOXP3 in the intestinal mucosa, so as to regulate native T cell differentiation into Treg cells; (3) moxibustion and acupuncture inhibit Th17 cell proliferation and the immune response and thus weaken the activity of effector T cells and control the intensity of the immune response through the stimulation of the expression of Treg cell and transcription factor FOXP3 in intestinal mucosa. As a result, the ratio of proinflammatory Th17 cells and anti-inflammatory Treg cells and their related molecules in intestinal mucosa tend to be restored and intestinal mucosal inflammation is alleviated.

Previous animal studies of our research group have also indicated that moxibustion and acupuncture can reduce intestinal inflammation in CD rats and promote the recovery of intestinal function. It is reported that moxibustion can decrease the levels of TNF-a and TNFR1, which are abnormally induced in CD rat colons, inhibit excessive apoptosis of colonic epithelial cells, and restore the colonic epithelial barrier [32, 33]. Furthermore, electronic acupuncture can regulate inflammatory signaling pathways in the intestine and immune-related signaling pathways in the spleen in rats with TNBS-induced colitis [34].

One of the limitations of this study is that the sample size is small. These results should be further validated with a larger sample size. Although the patients were followed up for 12 weeks after the treatment to assess their CDAI scores, no intestinal mucosa biopsies were taken to evaluate the long-term effect of moxibustion and acupuncture on the balance between Th17 and Treg cells. Future studies should combine clinical evaluation with microscopic and molecular studies of the intestinal mucosa to further elucidate the long-term efficacy of moxibustion and acupuncture.

## 5. Conclusion

In conclusion, this study shows that moxibustion and acupuncture can reduce the number of Th17 cells and downregulate the expression of Th17-related molecules IL-17 and ROR*γ*t and increase the number of Treg cells and upregulate the expression of Treg transcription factor FOXP3 in the intestinal mucosa of CD patients. This suggests that moxibustion and acupuncture relieve intestinal inflammation in CD patients by restoring the balance between Th17 and Treg cells, providing the basis for clinical application of treatment for CD.

## Figures and Tables

**Table 1:** Primer sequences used in RT-PCR.

Gene name	Primer	Sequence
IL-17	Forward	5′-TGAAGGCAGGAATCACAATC-3′
	Reverse	5′-CGGTTATGGATGTTCAGGTT-3′

ROR*γ*t	Forward	5′-ATGGAGCTCTGCCAGAATGA-3′
	Reverse	5′-TGCGGTTGTCAGCATTGTAG-3′

FOXP3	Forward	5′-CAGCGTGGTTTTTCTTCTCGGTATA-3′
	Reverse	5′-TGGTGAAGTGGACTGACAGAAAAG-3′
